# Laparoscopic robotic-assisted ileo-caecal resection with intracorporeal anastomosis in children with Crohn disease: initial experience of a paediatric center and surgical feasibility

**DOI:** 10.1007/s00383-024-05961-0

**Published:** 2025-01-17

**Authors:** Michela Cing Yu Wong, Giulia Rotondi, Stefano Avanzini, Serena Arrigo, Girolamo Mattioli

**Affiliations:** 1 Pediatric Surgery Department, IRCCS, Istituto Giannina Gaslini, Largo Gaslini 5, 16147 Genoa, Italy; 2 DINOGMI, University of Genoa, Genoa, Italy; 3 Pediatric Gastroenterology and Endoscopy Department, IRCCS, Istituto Giannina Gaslini, 16147 Genoa, Italy

**Keywords:** IBD, Robotic surgery, Pediatric CD, RICR

## Abstract

**Purpose:**

Pediatric-onset Crohn's disease (CD) presents with a more aggressive course than adults. Surgical treatment is still necessary in many patients. The laparoscopic technique for treating terminal ileal CD is deemed safe and feasible, with the advantage to perform an intra-corporeal anastomosis (ICA). The robotic platform facilitates ICA creation thanks to 3D-visualization, tenfold magnification and better dexterity. The aim of this study was to report our initial experience of robotic ileocecal resection (RICR) with ICA in children with CD.

**Methods:**

Six patients underwent RICR for CD. Patient characteristics, intraoperative details, and postoperative outcomes were collected. The surgical technique consisted in an intra-corporeal ICR with a stapled side-to-side ileo-colic anastomosis.

**Results:**

The mean age at surgery was 14.8 years. The mean operative time was 210.8 min. No intraoperative complications or conversions were recorded. Bowel function returned on postoperative day 3 and the mean hospital stay was 8 days. Two minor complications were treated conservatively and 1 major (anastomotic dehiscence) required reoperation.

**Conclusion:**

RICR is a safe and feasible technique in pediatric CD of terminal ileum. The robot offers advantages over other techniques for the precision of the suture, avoiding extracorporeal anastomosis. However, larger studies are needed to confirm these preliminary results.

## Introduction

Crohn's disease (CD) onset in childhood is characterized by a more extensive disease and more aggressive behavior, compared to adult [1]. The relevant advent of biological treatment has changed but not eliminated the need for surgery in many patients [1]. Surgery in pediatric CD is indicated as an alternative to medical therapy when a patient has active disease limited to a short segment despite optimized medical treatment and in children in prepubertal or pubertal stage if growth velocity for bone age is reduced over a period of 6 to 12 months in spite of optimized medical and nutritional therapy [2]. In children with localized ileocecal disease resistant to medical treatment, an elective surgical resection has been recommended as a viable treatment option, particularly for growth retarded prepubertal patients [3]. In patients with CD, surgical resection is not always curative and relapse, both at the area of anastomosis and at other sites, frequently occurs within 5 years post-surgery [4].

The laparoscopic approach for terminal ileal Crohn’s disease is reported to be safe, feasible, and without any increase in disease recurrence. One of the technical advantages of Minimally Invasive Surgery (MIS) is the ability to perform an intracorporeal anastomosis (ICA) [5]. There are data supporting the safety, feasibility, and short-term clinical benefits of MIS for CD; however, it is mainly for a laparoscopic approach. Robotic-assisted laparoscopic surgery (RALS) has been shown to have great merits in colorectal procedures, overcoming limitations of the laparoscopic approach, especially in the facilitation of an ICA creation [6]. In fact, as known, the robotic approach offers advantages over open and other minimally invasive techniques, including 3D visualization and tenfold magnification, which may enhance identification and preservation of critical anatomical structures. Moreover, robotic instrumentation effectively eliminates surgeon tremor and provides fine motion scaling. The pulley system at the tip of the robotic instrumentation results in wristed movement with additional degrees of freedom that is ideal for tissue dissection. Moreover, robot-assisted laparoscopic surgery (RALS) may allow the surgeon to perform an ICA and to shift the extraction site out from the midline, reducing the incisional hernia rate [6]. This technique also minimizes colon mobilization; in fact, in performing an ileocolic resection, the mobilization of the ascending colon could be limited to what is necessary for safe division of the bowel distal to the diseased segment [7]. There is limited published data examining the use of robotic assisted techniques in CD in adult literature, while no publications are found for the pediatric field.

The aim of the study was to report a single-center case series and to examine the feasibility and safety of Robotic ileo-caecal resection (RICR) with ICA in pediatric patients affected by CD, showing outcomes and complications.

## Materials and methods

The records of all patients aged less than 18 years, who underwent RICR for CD at our tertiary care children’s hospital, were obtained. The study period spanned from August 2015 to April 2016 and from December 2020 to July 2022, during which the robotic system was available at our center, encompassing a total duration of 27 months. Ethical committee approval was obtained on 18/10/2023, protocol n° 206/2023 – DB id 13,137.

Patient characteristics, age at surgery, intraoperative details, postoperative outcomes were collected and analyzed. The Clavien–Dindo classification was utilized to define the grade of complications [8]. Data concerning antibiotic therapy return to nutrition and first post-operative evacuation, blood transfusion, and length of hospital stay (LOS) were also gathered. Exclusion criteria were the lack of anastomosis and stoma fashioning proximal to the anastomosis. Descriptive statistics were reported as absolute frequencies and percentages. Mean, median and ranges were employed to describe semiquantitative and quantitative variables.

### Surgical technique

A perioperative Enhanced Recovery Gastrointestinal Surgery (ERAS) protocol is used for all the patients. All cases are discussed by a multidisciplinary team. Operative team is composed by a pediatric experienced surgeon in surgery for inflammatory bowel disease (IBD) at the robotic console, a robotic-trained surgeon and a resident at the patient side.

**Fig. 1:**
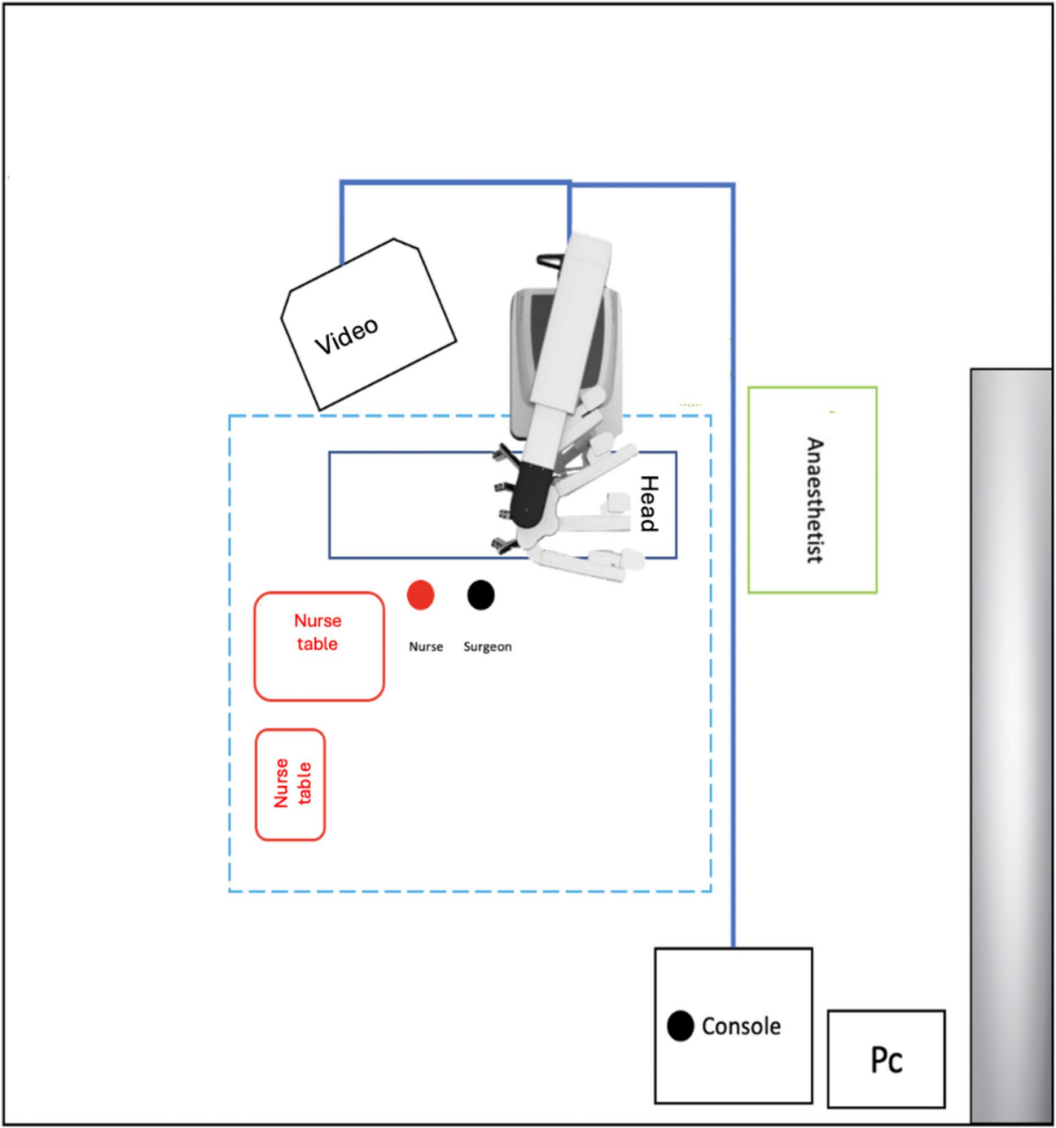
Operative room setting

The operating theatre and robotic Xi Da Vinci (Intuitive Surgical, Inc., Sunnyvale, CA, USA) technology setup are depicted in Fig. 1. Pre-operative intravenous antibiotics are administered at least 30 min before the incision.

**Fig. 2:**
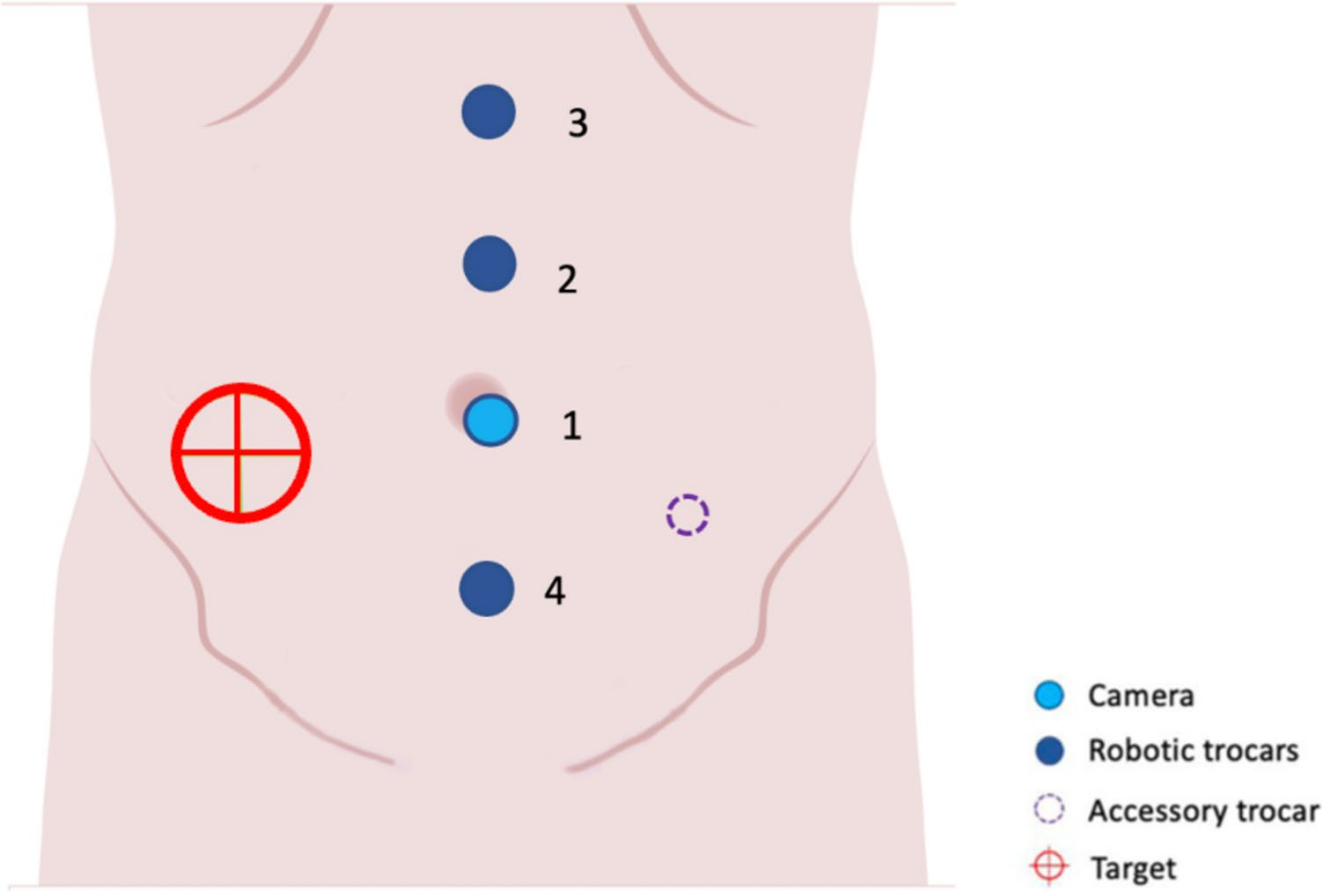
Robotic ports placement. Each trocar is placed 4–6 cm apart to prevent conflicts between robotic arms

The patient is placed in the supine position, in slight anti-Trendelenburg with the right side facing upward. Four ports are placed, on the xiphopubic line, under direct vision with the robotic camera positioned in the umbelicus. Each trocar is placed 4–6 cm apart to prevent conflicts between robotic arms (Fig. 2).

**Fig. 3:**
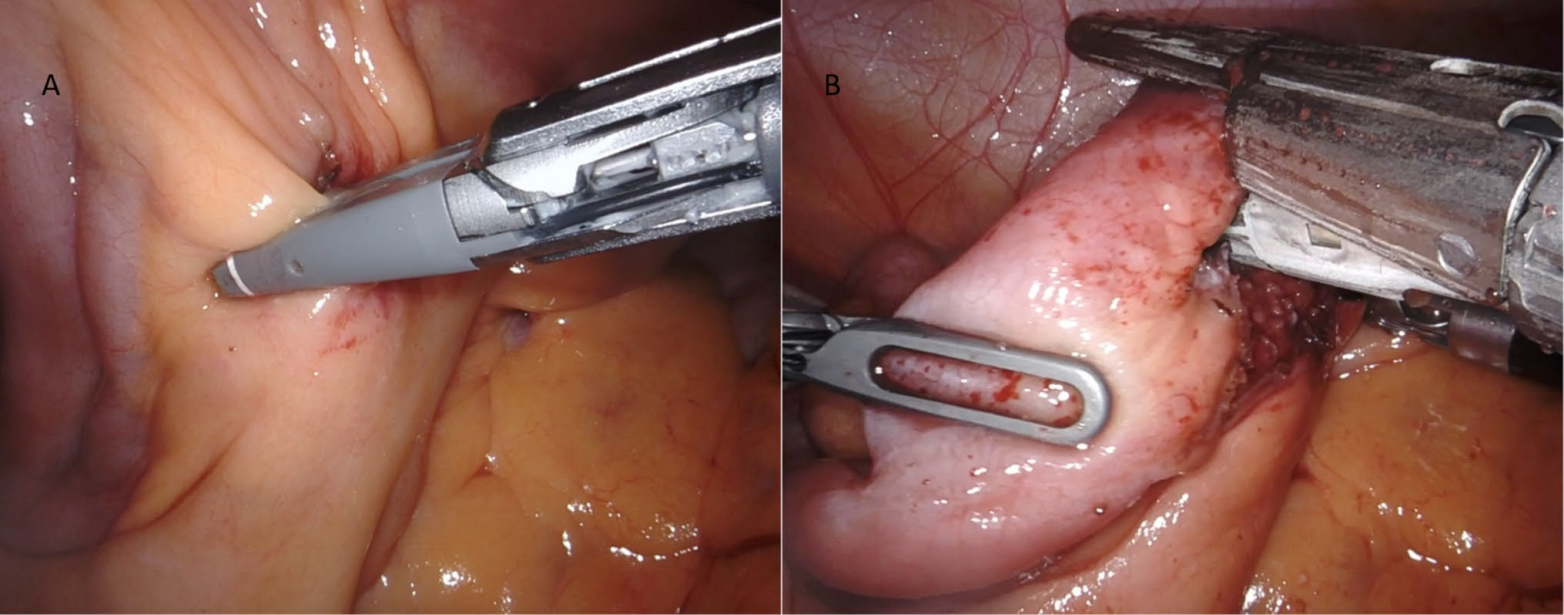
**A** Vessel sealing of the terminal ileum mesentery with a radiofrequency device for optimal bleeding control and creation of mesenteric window **B** Dissection of the ileum with a linear mechanical stapler at the site of healthy ileum, proximal to the stenotic ileal disease

**Fig. 4:**
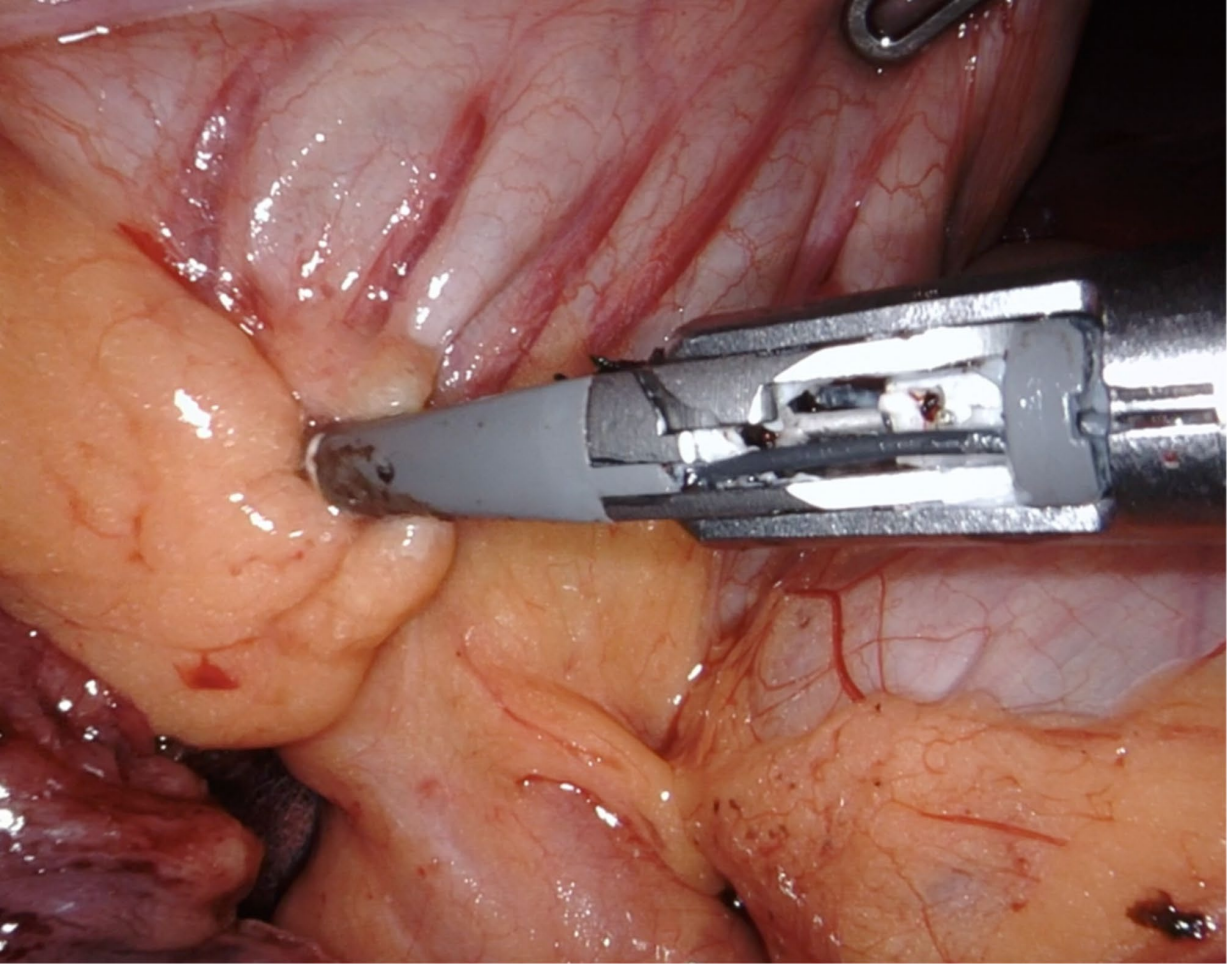
Vessel sealing of the ascending mesocolon with a radiofrequency device for optimal bleeding control and creation of mesocolon window

**Fig. 5:**
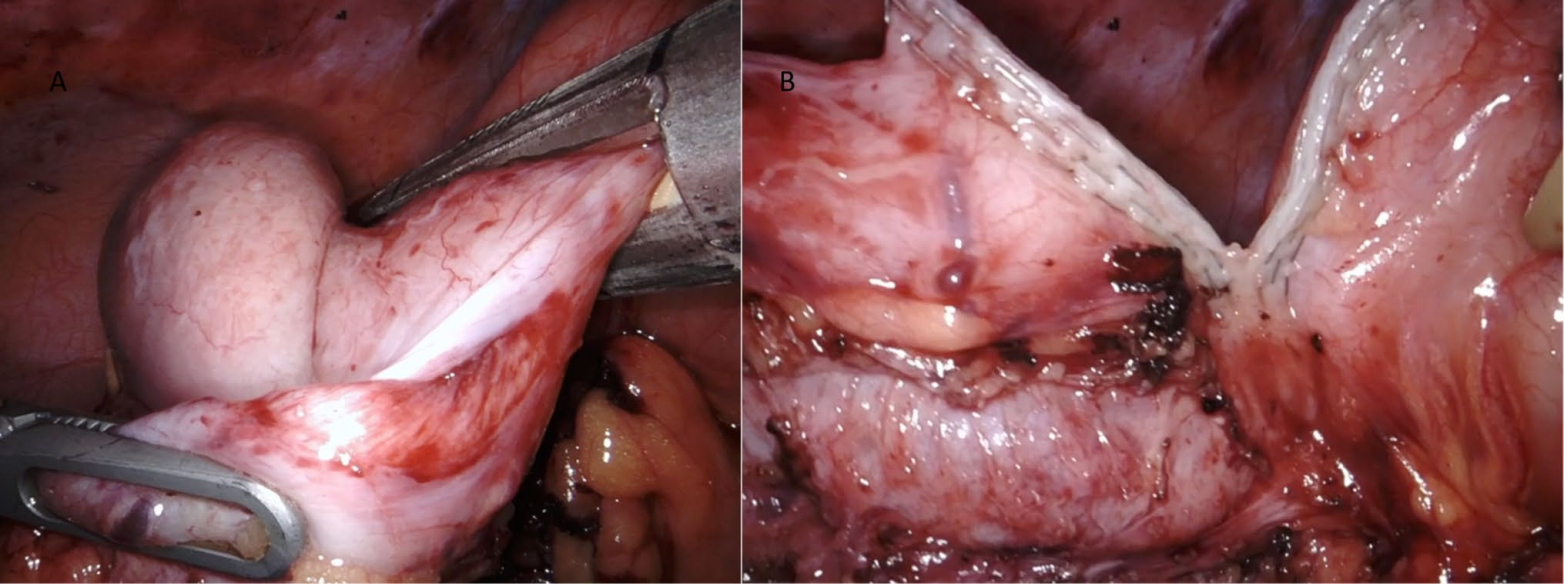
**A** Dissection of the colonic tissue not affected by disease with a linear mechanical stapler at the site of healthy right colon. **B** Dissection of the colonic tissue not affected by disease

**Fig. 6:**
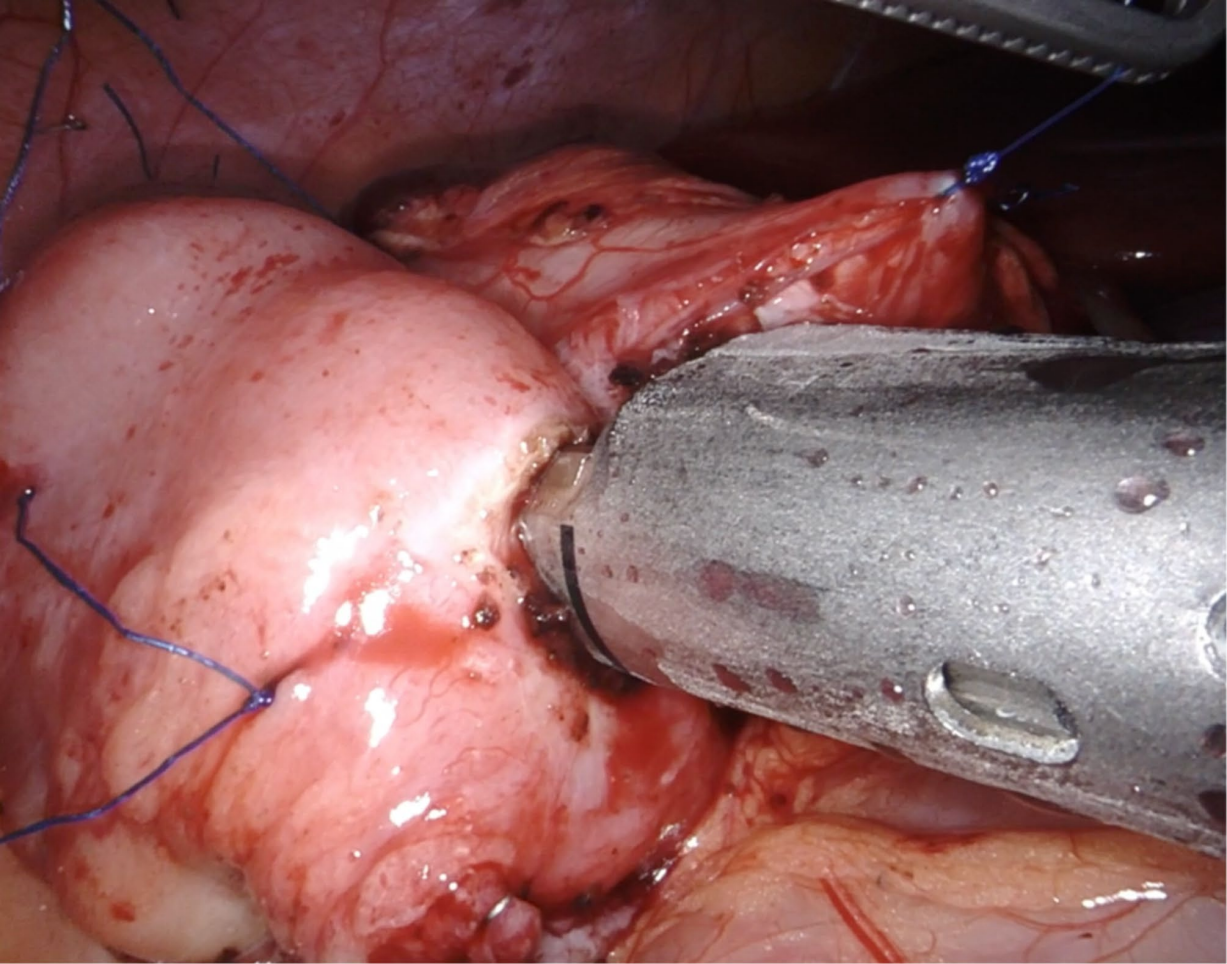
The terminal ileum and colon stumps are aligned isoperistaltically with stay sutures, resulting in a stapled ileocolic anastomosis

The ICR for CD consists in mobilizing and resecting the whole affected segments of bowel, determined by the presence of a clearly thickened ileal segment with a mesentery that is also macroscopically inflamed. The cecum and ascending colon are identified and mobilized through a lateral-to-medial dissection, ensuring direct visualization and protection of the right ureter, inferior vena cava, and duodenum. Once the affected bowel is sufficiently mobilized, ICR is performed intra-corporeally through division of mesentery and mesocolon with the use of a radiofrequency vessel sealer for optimal bleeding control (Fig. 3A-B, Fig. 4, Fig. 5A-B). The stumps of terminal ileum and ascending colon are aligned in a side-to-side manner in an isoperistaltic fashion with stay sutures, culminating in a stapled ileocolic ICA (Fig. 6). The intestinal opening after the side-to-side anastomosis is sutured with two continuous sutures. Vessel ligation, mesocolon division, and anastomosis are performed based on disease extension. The specimen can then be put in a specimen bag and removed via a Pfannenstiel incision or via a muscle splitting incision at the largest trocar site.

## Results

### Patient characteristics

A total of 6 paediatric patients, 2 males and 4 females were treated. Mean age at RICR was 14.8 years (median 15.6; range 11.2 -16.8). The mean weight was 48.4 kg (median 48.7; range 34.3 – 66), mean BMI was 19.3 (median 19.1; range 16**,**7 – 23.8). Diagnosis of CD was made at a mean age of 11.9 years (median 12.2; range 9.6 – 13.9).

All characteristics were summarized in Table 1.

**Table 1:** Patient features and operative data

Patient N°	Age at surgery (Years)	Sex	Weight (Kg)	Open Conversion	Intraoperative Complications	Postoperative Complications (Clavien–Dindo)						Surgical Time (min)	LOS (days)
						I	II	IIIa	IIIb	IV	V		
1	15.9	M	53	No	No							195	4
2	16.5	F	46.5	No	No	Fever						165	15
3	14.8	F	51	No	No	Fluid collection						205	8
4	16.8	F	40	No	No			Anastomotic dehiscence				230	16
5	11.2	F	34.3	No	No							295	8
6	15.4	M	66	No	No							175	5

### Perioperative results

Surgery was indicated for medically intractable CD. The patients in our cohort were initially treated with medical therapy, including corticosteroids immunomodulators and anti-TNF biologics. Surgical intervention was performed only on those patients who did not respond to medical treatment. The Pediatric Crohn's Disease Activity Index (PCDAI) at the time of surgery was > 30 in all patients.

Overall mean operative time was 210.8 min (median 200; range 165–295) with mean console time of 153.3 min (median 142.5; range 120–230). Lateral-to-lateral stapled ICA was performed for all patients. The median return to bowel function was post-operative day (POD) 3 (range 3–4). Children resumed oral feeding on the second/third post-operative day depending on clinical general conditions (median 3; range 2–4). The median LOS was on POD 8 (range 4 – 16). In the post-operative period, the patients' pain was managed with morphine infusion; only in one case an epidural catheter was placed. On the first post-operative day, the pain never exceeded a value of 6 on the NRS (Numerical Rating Scale). Upon discharge, the pain score was 0 for all patients.

### Complications

No intraoperative complication or conversions occurred. Three (50%) postoperative complications happened during the study period, shown in Table 1. Two patients developed minor complications treated conservatively (Clavien–Dindo II), namely transient fever and fluid collection treated with antibiotics. One patient had an anastomotic dehiscence, requiring reoperation with ileostomy fashioning, at another hospital (Clavien–Dindo IIIB). No life-threating complication or death occurred.

## Discussion

The most commonly performed surgical procedure for CD in pediatric patients is ICR for terminal ileal disease. In 2003, Dutta et al. reported that laparoscopic resection of CD in children was safe and effective [9].

The advantages associated to laparoscopy are well established in the literature: fewer complications, enhanced recovery and shorter length of stay compared to open approach [10, 11]. Robotic surgery has the same advantages as laparoscopic surgery, with the additional advantage of working in difficult anatomical conditions and in confined spaces [12].

Patients with CD are challenging for the surgeon, especially in younger patients than in adults, due to the more aggressive nature of the disease. The hard-to-manipulate thickened and friable mesentery can result in high blood loss, adhesions to the peritoneal surface and other organs, abscesses, and fistulas [7]. Better visualization, instrumentation, tremor reduction, and dexterity that the robotic approach offers enable surgeons to overcome these difficulties. Emerging data on robotic surgery on adult patients with CD have shown a slightly faster return to bowel function, compared to laparoscopic approach [6, 13–15]. In our group, all patients had rapid return of bowel movements. Several authors in literature state that a robotic approach can allow the surgeon to perform an intracorporeal anastomosis, which has been associated with decreased rates of postoperative ileus [16–18].

Ileocolic anastomosis can be accomplished with an ICA or extracorporeal anastomosis (ECA) technique. However, due to its technical challenges, ICA is not widely adopted in conventional laparoscopy, while the robotic platform enables easier suturing with wristed instruments which facilitates construction of ICA [5]. Kelley et al. described a series of 144 adult patients undergoing right hemicolectomy for cancer (87 laparoscopic, 27 robotic). Patients who underwent robotic ICA had shorter return to bowel function, lower rates of postoperative ileus, lower overall postoperative complications, less blood loss, lower narcotic use, but longer operative time compared to laparoscopy with extracorporeal anastomosis [19]. Robotic surgery can contribute to more minimizing tissue trauma, allowing faster postoperative recovery and potentially fewer long-term complications [20].

Robotic approach does not require mobilization of the ascending colon more than is necessary for resection of the diseased segment; the ascending colon can be left in place, reducing adhesion formation and allowing the duodenum to remain in the retroperitoneum protected by the right colon and its mesentery [7]. In CD, most fistulas to the duodenum originate from recurrent disease in the terminal ileum after previous ICR [21]. Avoiding colonic mobilization reduces the rate of this kind of complication.

Moreover, ICA compared with extracorporeal anastomoses, can reduce number of postoperative complications, anastomotic leak rate and surgical site infections [22–24]. In their study, Aydinli et al. suggested that ICA is usually not practiced in conventional laparoscopy [6] for the technical difficulty. The improved ease of suturing offered by robotic platforms may help overcome this limitation and facilitate the construction of ICA. Potential benefits from this technique include improved cosmetics, reduced postoperative pain, reduced risk of incisional hernia occurrence, and faster recovery of bowel function.

Intra-abdominal postoperative septic complications are the most feared complications of surgery for CD. On adult patients, RICR was associated with a significant reduction of intra-abdominal septic complications and decreased severe complications (Clavien–Dindo classification ≥ III) [25]. In our series, among patients who reported complications, only 1 (16,6%) had a severe complication (Clavien–Dindo ≥ III), showing anastomotic dehiscence 12 days after the operation, needing an open reoperation in another hospital. The patient had had a pre-operative diffuse peritoneal inflammation, with a fluid collection of 38 mm close to the terminal ileum, associated with a debilitated health status (BMI 16.7). Analyzing more in-depth this complication, we understand that the selection and timing of operation should always be carried out with the utmost care, providing extra time for preparation when necessary.

According to the literature, early postoperative complications in children occur in up to 30% of cases during surgery on the small intestine or in the ileocecal region. IBD surgery is also subjected to a higher complication rate compared to similar surgeries for other diseases. Preoperative medical treatment, especially biologic treatments may contribute to the development of complications [26–28].

In the end, the use of the robotic platform has some known limitations; these include higher costs, longer operative time, especially in center where few robotic operations are performed, and a different learning curve. Actually, the equipment costs for robotic surgery have come down in recent years, in particular thanks to the increasing diffusion of this system and to the increased competition. In fact, recently, new robotic platforms entered the market aiming to reduce costs and improve the access to robotic surgery [29]. The learning curve for robotic surgery has been shown to be faster, compared to laparoscopic approach [30].

## Conclusion

To our knowledge, this is the first paper reporting on the application of RICR in pediatric population. The limitation of the study is the small patient sample, and we think that a broader sample is required for validation of our results. Furthermore, in view of the novel surgical approach, it will be important to evaluate long-term follow-up and recurrence of disease.

According to our experience, RICR is a safe and feasible option in pediatric patients with CD and RALS offers advantages over other techniques for the precision of anastomosis. Larger studies and a long-term follow-up are needed to confirm our findings. Finally, improving robotic surgical training and establishing standardized protocols for this technique also would add strength to this study.

## Data Availability

No datasets were generated or analysed during the current study.
